# Persistent Redox Imbalance in Post-COVID-19 Syndrome: Lipid Peroxidation, Antioxidant Enzyme Depletion, and Reduced Nitric Oxide Availability in an Amazonian Population

**DOI:** 10.3390/antiox15081013

**Published:** 2026-08-14

**Authors:** Renata Cunha Silva, Juarez Antônio Simões Quaresma, Bruno Kenji Hosoda Mineshita, Maírha Eduarda Alcântara Costa, Verônica Myrna Cordeiro Reis, Camile Irene Mota da Silva, Rebeca Fontenele Pinheiro, Lucas Gabriel Viana Barbosa, Kathellen Joane Silva Miranda, Elem Cristina Rodrigues Chaves, Daniel Carvalho de Menezes, Jofre Jacob da Silva Freitas, Robson José de Souza Domingues, Pedro Fernando da Costa Vasconcelos, Luiz Fábio Magno Falcão

**Affiliations:** 1 Integrated Laboratory for Research and Care in Infectious and Sequelae Diseases in the Amazon, State University of Pará, Belém 66095-662, PA, Brazil; renata.csilva@aluno.uepa.br (R.C.S.);; 2 Department of Pathology, Escola Paulista de Medicina, Federal University of São Paulo, São Paulo 04023-062, SP, Brazil; juarez.quaresma@unifesp.br; 3 Integrated Laboratory for Research and Care in Infectious and Sequelae Diseases in the Amazon, Federal University of Pará, Belém 66075-110, PA, Brazil; 4 Integrated Laboratory for Research and Care in Infectious and Sequelae Diseases in the Amazon, Federal Rural University of the Amazon, Belém 66077-830, PA, Brazil; 5 Molecular Biology Laboratory (LABIOMOL), Institute of Biological Sciences, Federal University of Pará, Belém 66075-110, PA, Brazil; rebeca.pinheiro@icb.ufpa.br; 6 Laboratory of Natural Products Chemistry, Federal University of Pará, Belém 66075-110, PA, Brazil; 7 Laboratory of Natural Products Chemistry, Metropolitan University Center of the Amazon (UNIFAMAZ), Belém 66053-000, PA, Brazil; 8 Center for Pre-Clinical Studies of the Amazon, State University of Pará, Belém 66095-662, PA, Brazil; 9 Laboratory for Didactic Development and Research in Health Education (LADDIPES), State University of Pará (UEPA), Belém 66095-662, PA, Brazil; 10 Arbovirology Laboratory, Evandro Chagas Institute, Ananindeua 67030-000, PA, Brazil; pedro.vasconcelos@uepa.br

**Keywords:** post-COVID-19 syndrome, long COVID, oxidative stress, lipid peroxidation, antioxidant enzymes, nitric oxide

## Abstract

Post-COVID-19 syndrome (PCS) can persist even after mild acute illness, and oxidative stress has been proposed as a central mechanism for this persistence, although it remains poorly studied in the Amazon. This study characterized redox imbalance in patients with PCS through a combined analysis of lipid peroxidation, antioxidant defenses, and nitric oxide bioavailability. In a cross-sectional observational design, 85 patients with PCS and 53 healthy controls were evaluated, with determination of TBARS, total antioxidant capacity (ABTS), erythrocyte SOD, CAT, and GPx activity, plasma nitric oxide levels, and subgroup analysis according to disease duration (≤6, 6–12, and >12 months). Patients with PCS showed significantly higher TBARS concentrations and lower nitric oxide, ABTS, SOD, CAT, and GPx levels than controls, with predominantly large effect sizes. Lipid peroxidation correlated positively with HDL-c levels, and none of the markers differed significantly among the symptom duration subgroups. The findings indicate that PCS is associated with a persistent redox imbalance, marked by continuous lipid oxidative damage and sustained depletion of enzymatic and non-enzymatic antioxidant defenses, with a vascular component suggested by reduced nitric oxide levels, regardless of the time elapsed since symptom onset.

## 1. Introduction

Post-COVID-19 syndrome (PCS), also known as long COVID, is prominent among the late consequences of SARS-CoV-2 infection. A proportion of infected individuals continue to experience symptoms for months after the acute phase, even after mild disease [1,2,3,4]. Chronic fatigue, reduced exercise tolerance, cognitive changes, muscle pain, and functional limitations are among the most frequently reported manifestations, suggesting that the syndrome extends beyond the initial respiratory involvement and affects multiple systems [2,5,6].

Recent studies have shown that patients with PCS exhibit inflammatory and metabolic alterations that persist over time. Among the most discussed mechanisms, oxidative stress stands out, characterized by increased production of reactive oxygen species (ROS) and decreased antioxidant capacity [7,8,9]. Mitochondrial alterations, prolonged inflammation, and endothelial injury appear to sustain this state after acute SARS-CoV-2 infection [10].

Biomarkers such as malondialdehyde (MDA) are associated with lipid peroxidation and persistent cellular damage, whereas decreased activity of antioxidant enzymes such as superoxide dismutase (SOD), catalase (CAT), and glutathione peroxidase (GPx) indicates impairment of the body’s antioxidant defenses [11,12,13]. Alterations in nitric oxide production have also been observed in post-SARS-CoV-2 patients, suggesting that nitrosative stress and endothelial dysfunction may be involved in the persistence of post-COVID-19 clinical manifestations [9].

Although recent evidence indicates that oxidative stress plays a significant role in the pathophysiology of post-COVID-19 syndrome [3,13,14], studies investigating the continuity of these alterations over time and their relationship with metabolic and vascular changes are scarce [15,16]. Moreover, most of the primary studies synthesized in recent reviews have been conducted in North American, European, and Asian populations [1,6], whereas data from the Brazilian Amazon remain limited. This gap is particularly significant, as environmental, nutritional, socioeconomic, and epidemiological aspects specific to the region may affect both the inflammatory response and redox balance after SARS-CoV-2 infection.

In addition, investigations that have jointly analyzed biomarkers of oxidative damage, enzymatic and non-enzymatic antioxidant defenses, and nitric oxide bioavailability in patients with long COVID remain scarce [3,13,14]. The persistence of these alterations over prolonged intervals after infection, as well as the extent to which they are related to endothelial dysfunction and the metabolic changes referred to in this condition, remains uncertain [9,15,17]. Thus, the mechanisms underlying redox imbalance in post-COVID-19 syndrome have not yet been fully elucidated, particularly in underrepresented groups such as the Amazonian population of Brazil.

Therefore, this study aimed to characterize the redox imbalance associated with post-COVID-19 syndrome in an Amazonian population through the integrated analysis of biomarkers of lipid peroxidation, antioxidant capacity, and nitric oxide bioavailability. In addition, we sought to investigate the relationship between these factors and metabolic alterations and the temporal persistence of the syndrome. We hypothesized that individuals with PCS present a persistent increase in oxidative damage associated with reduced antioxidant defenses and altered nitric oxide production, which contributes to the persistence of metabolic alterations and prolonged clinical manifestations after infection.

## 2. Materials and Methods

### 2.1. Study Population

This observational, cross-sectional, analytical–descriptive study was approved by the Research Ethics Committee for Human Subjects of the State University of Pará (protocol no. 4.252.664). All participants signed an informed consent form, and the research was conducted in accordance with the principles of the Declaration of Helsinki.

The sample consisted of adults (≥18 years) of both sexes, selected by convenience between March 2020 and March 2023, following voluntary enrollment in a clinical care initiative for patients with long COVID in the metropolitan region of Belém, state of Pará, in the Brazilian Amazon.

A total of 145 individuals voluntarily enrolled in the study. The inclusion criteria were as follows:

(a) Confirmation of symptomatic infection during the acute phase by SARS-CoV-2 using real-time reverse transcription polymerase chain reaction (rRT-PCR);
(b) A positive result for SARS-CoV-2–specific IgM (criterion adopted prior to vaccination);
(c) The manifestation of at least one persistent COVID-19 symptom, such as fatigue, dyspnea, cough, chest pain, muscle pain or weakness, headache, insomnia, visual disturbances, tremors, loss of balance, lower-limb edema, arthralgia, and/or changes in taste and/or smell, lasting at least four weeks (30 days) after the onset of acute symptoms, not attributable to another cause, in accordance with the World Health Organization (WHO) criteria for long COVID [18].

The exclusion criteria were as follows: patients with autoimmune diseases (*n* = 12), individuals with diabetes mellitus (*n* = 47), and severe cases of acute SARS-CoV-2 infection (*n* = 1), according to the WHO criteria [18].

Thus, after applying these criteria, the final sample consisted of 85 participants who met criteria (a), (b), and (c) and were diagnosed with post-COVID-19 syndrome [18]. Biological samples were collected at three time points: ≤6 months, >6–12 months, and >12 months.

For comparative purposes, blood samples from 53 healthy controls with no history of SARS-CoV-2 infection were obtained from a previous study by the same research group (protocol no. 2.629.048) collected in 2018. Importantly, the redox biomarker assays for this control cohort (TBARS, NO, TEAC, SOD, CAT, and GPx) were performed in 2018, at the time of sample collection and processing, using the same standardized protocol and laboratory methodology later applied to the PCS group; raw biological material was not re-analyzed years after collection. This strategy of using a pre-pandemic biobank cohort as a historical control, with assays performed contemporaneously with collection, follows an approach similar to that adopted in other post-COVID-19 redox biomarker studies [3].

Patients with long COVID and control group individuals with a prior diagnosis of neurodegenerative or neuroinflammatory disorders, such as chronic fatigue syndrome [19], Parkinson’s disease, Alzheimer’s disease, multiple sclerosis, and stroke, were excluded. Patients with systemic autoimmune diseases or similar conditions, such as diabetes mellitus, psoriasis, systemic lupus erythematosus, rheumatoid arthritis, inflammatory bowel disease, and scleroderma, and pregnant women were excluded from the study [14].

### 2.2. Sample Collection and Processing

Demographic, clinical, and laboratory data were also collected. Venous blood samples were obtained after an 8–12 h fasting period using sterile vacuum tubes containing ethylenediaminetetraacetic acid (EDTA) for analyses related to glucose and oxidative metabolism. After collection, the samples were centrifuged at 2500× *g* for 10 min in a Daiki 80-2B centrifuge (Jiangsu Jinyi Instrument Technology Co., Ltd., Jintan, Changzhou, Jiangsu, China).

For the lipid profile, blood was collected in tubes containing a clot activator to obtain serum samples. The samples were allowed to stand until coagulation was complete and then centrifuged under the same conditions to separate the serum. The obtained aliquots were immediately used for biochemical analyses.

Serum concentrations of total cholesterol, high-density lipoprotein (HDL-c), triglycerides, and glucose were determined using the enzymatic colorimetric method with validated commercial kits (Wiener Laboratorios S.A.I.C., Rosario, Argentina), according to the manufacturer’s instructions. The results were expressed in mg/dL.

After centrifugation, plasma and erythrocytes were stored at −80 °C for further analyses. Before freezing, erythrocytes were washed twice with 0.9% saline solution to remove residual plasma and subsequently used to determine the activity of the antioxidant enzymes catalase (CAT), glutathione peroxidase (GPx), and superoxide dismutase (SOD).

Plasma was used for quantification of total antioxidant capacity (ABTS), thiobarbituric acid reactive substances (TBARS), and nitric oxide levels. Analyses related to oxidative metabolism were performed using a CLARIOstar microplate spectrophotometer (BMG LABTECH, Ortenberg, Germany), with the exception of catalase activity, which was assessed using an Ultrospec 7500 cuvette spectrophotometer (Biochrom Ltd., Cambridge, UK).

### 2.3. Determination of Antioxidant Levels

#### 2.3.1. Determination of Catalase Activity

Catalase (CAT) activity was assessed in erythrocyte hemolysate prepared by cell lysis using distilled water. Enzymatic activity was measured according to the method described by Aebi [20], which determines the rate of hydrogen peroxide (H_2_O_2_) decomposition at 240 nm, monitored using a spectrophotometer. Measurements were performed at 20 °C, the standard assay temperature recommended in the original protocol, which exhibits low temperature dependence over the 0–37 °C range. CAT activity was expressed in units per milligram hemoglobin (U/mg Hb).

#### 2.3.2. Determination of Glutathione Peroxidase (GPx) Activity

Glutathione peroxidase (GPx) activity was determined in erythrocyte lysates using the method described by Flohé and Günzler [21]. To avoid interference from hemoglobin oxidation in the lysates, KCN was added (Sigma-Aldrich, St. Louis, MO, USA) to the reaction medium to convert hemoglobin to methemoglobin and prevent the overestimation of enzymatic activity. Measurements were performed at 37 °C.

The assay is based on the reduction of tert-butyl hydroperoxide (t-BuOOH) (Sigma-Aldrich, St. Louis, MO, USA), accompanied by the oxidation of reduced glutathione (GSH) to oxidized glutathione (GSSG) catalyzed by GPx. Glutathione reductase (GR) regenerates GSH using NADPH, and the decrease in absorbance is monitored at 340 nm. Thus, the rate of NADPH oxidation is directly proportional to GSSG formation and, therefore, to GPx activity in the sample. Enzymatic activity was expressed in units per milligram of Hb.

#### 2.3.3. Determination of Superoxide Dismutase (SOD) Activity

Samples intended for SOD activity determination were hemolyzed by mixing blood with ice-cold distilled water, chloroform, and ethanol (Sigma-Aldrich, St. Louis, MO, USA), which is a procedure used to remove hemoglobin. Total SOD activity was quantified by inhibition of cytochrome c reduction mediated by the superoxide anion generated in the xanthine/xanthine oxidase enzymatic system (Sigma-Aldrich, St. Louis, MO, USA) at pH 7.8 and 25 °C, as described by Flohé and Otting [22].

The amount of xanthine oxidase used was adjusted to produce a cytochrome c reduction rate of 0.025 A.U./min at 550 nm wavelength. One unit of SOD activity was defined as the amount of enzyme required to inhibit the rate of cytochrome c reduction by 50%. Enzymatic activity was expressed in units per milligram of Hb.

### 2.4. Determination of Total Antioxidant Capacity (ABTS)

Plasma antioxidant capacity was assessed using spectrophotometry, according to the method described by Re et al. [23]. This method is based on the reaction between the ABTS•+ radical (2,2′-azino-bis-3-ethylbenzothiazoline-6-sulfonic acid diammonium) (Calbiochem, Merck KGaA, Darmstadt, Germany) and potassium persulfate (K_2_S_2_O_8_), generating a solution with an absorbance between 0.680 and 0.720 at 734 nm. Antioxidants present in the plasma reduce the ABTS•+ radical to ABTS, proportionally decreasing the absorbance of the solution.

The total antioxidant capacity was determined from a standard curve constructed using Trolox (Calbiochem, Merck KGaA, Darmstadt, Germany), a water-soluble analog of vitamin E. The results were expressed in mmol/L of Trolox.

### 2.5. Determination of Pro-Oxidant Levels

#### 2.5.1. TBARS (MDA) Assessment

The concentration of thiobarbituric acid reactive substances (TBARSs) was determined using spectrophotometry [24,25]. This method is based on the colorimetric reaction between lipid peroxidation products, particularly malondialdehyde (MDA), and 0.8% thiobarbituric acid (TBA) (Sigma-Aldrich, St. Louis, MO, USA), carried out under acidic conditions and heating between 90 and 100 °C. The TBARS concentration was expressed in µmol/L. As the TBARS assay is a widely used indirect (colorimetric) indicator of MDA and may also react with other non-lipid carbonyl compounds, results are reported throughout the manuscript as TBARS (MDA) to reflect this distinction. A linear standard curve for MDA was constructed using serial dilutions of a malondialdehyde bis (dimethyl acetal) standard (Sigma-Aldrich, St. Louis, MO, USA), yielding the equation y = 0.0002x + 0.0067 (R^2^ = 0.9998), where x is MDA concentration (µmol/L) and y is absorbance. Samples were not separated by high-performance liquid chromatography (HPLC) prior to the colorimetric reaction; consequently, as noted above, the TBARS values reported here reflect total thiobarbituric acid-reactive material rather than MDA in isolation, and this lack of chromatographic separation is acknowledged as a limitation of the assay.

#### 2.5.2. Indirect Assessment of Nitric Oxide

Indirect determination of nitric oxide (NO) was performed by measuring plasma nitrite/nitrate (NOx) levels using the Griess test. Samples were first deproteinized with 20 μL of 30% zinc sulfate (ZnSO_4_) added to 400 μL of serum, which was then centrifuged at 2000 rpm for 15 min.

Next, 50 μL of the supernatant and 50 μL of vanadium chloride (8 mg/mL) (Sigma-Aldrich, St. Louis, MO, USA), which was used to reduce nitrate to nitrite, were pipetted. After adding 50 μL of Griess reagent, the samples were incubated at 37 °C for 20 min. Absorbance was measured at 550 nm [26], and the results were expressed in μmol/L.

### 2.6. Statistical Analysis

Categorical variables were expressed as absolute and relative frequencies (n and %) and analyzed using the chi-square test. Continuous variables are presented as median and interquartile range [IQR: Q1–Q3]. The normality of the data distribution was assessed using the Shapiro–Wilk test. Based on the results obtained, comparisons between the two independent groups were performed using the non-parametric Mann–Whitney U test. For comparisons among the three long COVID duration subgroups (G1: ≤6 months; G2: >6 to 12 months; G3: >12 months), the Kruskal–Wallis test was used, followed by Dunn’s post hoc test with Bonferroni correction for multiple comparisons. All tests were two-tailed, with a significance level of *p* < 0.05. Spearman or Pearson correlation coefficients were used to assess the correlations between the lipid profiles and pro-oxidant markers according to the data distribution. All statistical analyses were performed using GraphPad Prism version 11.0.1 (GraphPad Software, San Diego, CA, USA).

## 3. Results

### 3.1. Demographic, Laboratory, and Clinical Characteristics

The characteristics of the healthy control (HC) and post-COVID-19 syndrome (PCS) groups are presented in Table 1. As continuous variables were non-normally distributed (Shapiro–Wilk test), they are presented as the median (Q1–Q3), with between-group comparisons performed using the Mann–Whitney U test, consistent with Section 2.6. Patients with PCS were significantly younger than controls 49 (42–60) vs. 55 (47–62) years; *p* = 0.035. Regarding sex, a significantly higher proportion of women was observed in the PCS group than in the HC group (77.65% vs. 45.28%; *p* = 0.0002). Regarding biochemical parameters, the PCS group showed significantly higher total cholesterol 201 (179–235) vs. 174 (157–194) mg/dL; *p* = 0.0001 and LDL-c 123 (101–153) vs. 105.5 (86–119.25) mg/dL; *p* = 0.0012 than the HC group. HDL-c 50 (44–59) vs. 52 (46–57) mg/dL; *p* = 0.423, fasting glucose [94 (88–100) vs. 95 (87–105) mg/dL; *p* = 0.887], and triglyceride concentrations 124 (84–175) vs. 134 (94–170) mg/dL; *p* = 0.506, did not differ significantly between groups. The most prevalent symptoms in the PCS group were fatigue (83.33%), headaches (54.76%), and insomnia (46.43%).

### 3.2. Serum Levels of Oxidative Stress Biomarkers and Erythrocyte Antioxidant Enzyme Activity in Patients with Post-COVID-19 Syndrome

**Figure 1:**
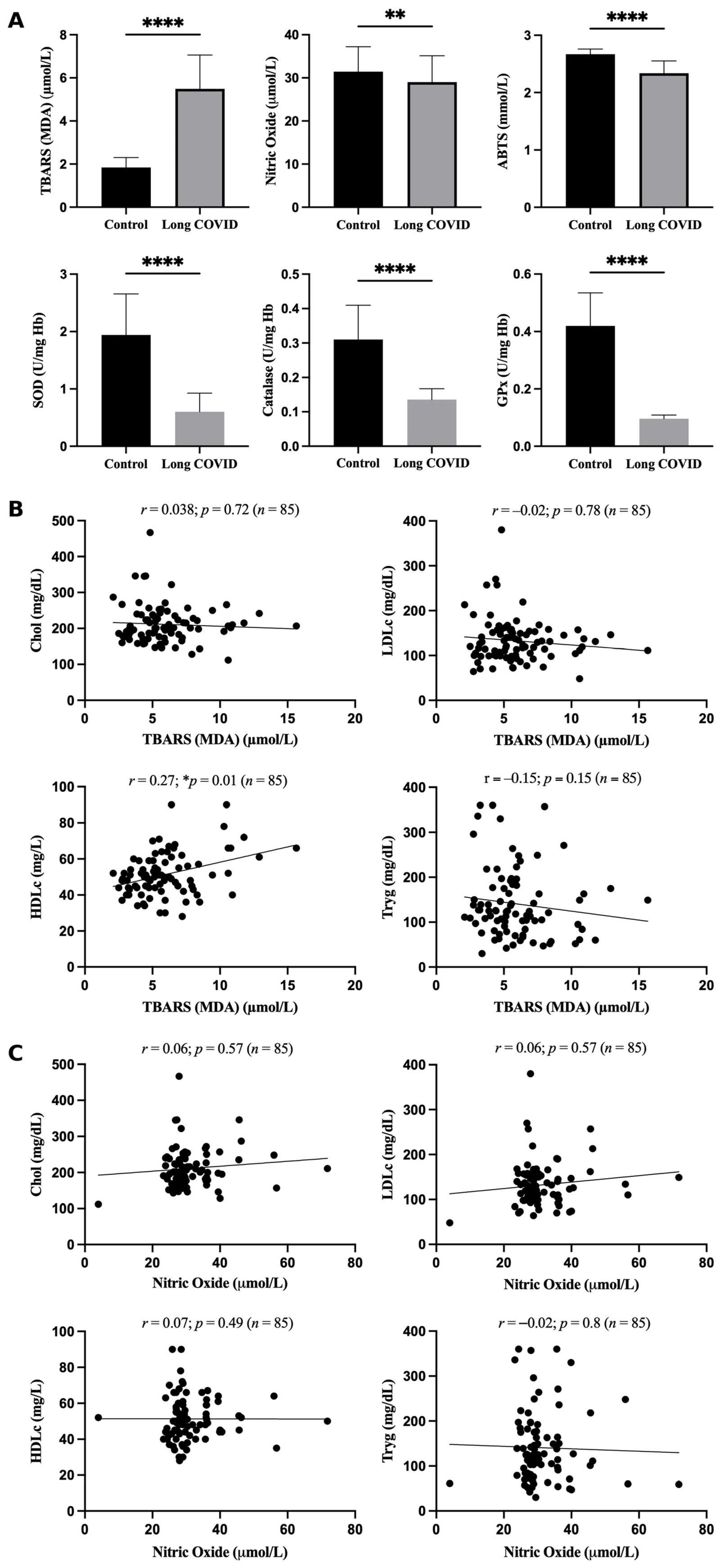
Oxidative stress biomarkers and their association with lipid profile in patients with post-COVID-19 syndrome (PCS) and healthy controls (HCs). (**A**) Levels of oxidative stress markers in healthy individuals and patients with post-COVID-19 syndrome. Pro-oxidant marker levels were significantly elevated in patients with post-COVID-19 syndrome, whereas total antioxidant capacity and antioxidant enzyme activity were reduced compared to healthy controls. Statistical analyses were performed using the Mann–Whitney U test. ** *p* < 0.01 and **** *p* < 0.0001 indicate statistically significant differences between the compared groups. (**B**) The association between malondialdehyde (MDA) levels and lipid profile in patients with post-COVID-19 syndrome. The scatter plots show the associations between MDA and total cholesterol (Chol), LDL-c, HDL-c, and triglyceride (Tryg) levels. A significant positive correlation was observed between MDA and HDL-c levels (rs = 0.28; *p* = 0.010 *). No significant correlations were observed with total cholesterol (rs = 0.04; *p* = 0.728), LDL-c (rs = −0.03; *p* = 0.789), or triglyceride levels (rs = −0.15; *p* = 0.158). (**C**) The correlation between nitric oxide (NO) levels and lipid profile in patients with post-COVID-19 syndrome. The scatter plots show the associations between NO and total cholesterol (Chol), LDL-c, HDL-c, and triglyceride (Tryg) levels. No significant correlations were observed between NO and total cholesterol, LDL-c, HDL-c, or triglyceride levels (*n* = 85). Analyses in (**B**,**C**) were performed using Spearman’s correlation coefficient. In (**B**,**C**), each black dot represents an individual patient with post-COVID-19 syndrome (*n* = 85), and the solid line indicates the linear regression fit; healthy controls were not included in the correlation analyses. * *p* < 0.05. Statistical significance was set at *p* < 0.05.

Patients with post-COVID-19 syndrome (PCS) showed significantly higher TBARS concentrations than healthy controls (HCs) median (IQR): 5.490 (4.330–6.950) vs. 1.846 (1.372–2.193) μmol/L MDA; *p* < 0.0001; r = 0.917, 95% CI: 0.818–0.990, indicating a marked increase in lipid peroxidation in the PCS group, with a large effect size. In contrast, plasma nitric oxide (NO) levels were significantly lower in the PCS group 29.01 (27.03–34.66) vs. 31.45 (28.79–36.99) μmol/L; *p* = 0.0076; r = −0.269, 95% CI: −0.450 to −0.069, although with a small effect size. Erythrocyte antioxidant enzyme activity was significantly reduced in the PCS group: superoxide dismutase (SOD) 0.600 (0.300–0.920) vs. 1.940 (1.270–2.650) U/mg Hb; *p* < 0.0001; r = −0.786, 95% CI: −0.886 to −0.662; catalase (CAT) 0.136 (0.111–0.164) vs. 0.310 (0.200–0.410) U/mg Hb; *p* < 0.0001; r = −0.587, 95% CI: −0.748 to −0.406; and glutathione peroxidase (GPx) 0.097 (0.085–0.109) vs. 0.420 (0.360–0.530) U/mg Hb; *p* < 0.0001; r = −1.000, the marker with the largest effect size. The total antioxidant capacity (TEAC/ABTS) was also significantly reduced in the PCS group compared to that in the HC group 2.340 (2.280–2.550) vs. 2.670 (2.620–2.760) mmol/L; *p* < 0.0001; r = −0.897, 95% CI: −0.952 to −0.828, with a very large effect size (Figure 1A).

Correlation analysis between TBARS levels and lipid profiles in the PCS group revealed no significant associations with total cholesterol (rs = 0.04; *p* = 0.728; *n* = 85), LDL-c (rs = −0.03; *p* = 0.789; *n* = 85), or triglycerides (rs = −0.15; *p* = 0.158; *n* = 85). However, there was a significant positive correlation between MDA and HDL-c (rs = 0.28; *p* = 0.010; *n* = 85), indicating that higher MDA levels were associated with higher HDL-c levels in the PCS group (Figure 1B).

Correlation analysis between NO levels and lipid profiles in the PCS group, including all participants (*n* = 85), revealed no significant associations with total cholesterol (rs = 0.06; *p* = 0.579), LDL-c (rs = 0.06; *p* = 0.575), HDL-c (rs = 0.07; *p* = 0.498), or triglycerides (rs = −0.03; *p* = 0.812) (Figure 1C).

To investigate whether the time elapsed since symptom onset influences the redox profile of patients, participants with post-COVID-19 syndrome were redistributed into three subgroups according to long COVID duration, G1 (≤6 months), G2 (>6–12 months), and G3 (>12 months), in accordance with the World Health Organization criteria (WHO, 2021) [18]. The sample sizes per subgroup were G1 = 12, G2 = 21, and G3 = 52 (*n* = 85) for all evaluated markers. Data are presented as the median (Q1–Q3), and comparisons among the three subgroups were performed using the Kruskal–Wallis test with Dunn’s post hoc test corrected by Bonferroni correction (Table 2).

None of the evaluated oxidative stress biomarkers showed statistically significant differences among the subgroups defined by long COVID duration (Table 2). Plasma nitric oxide (NO) levels did not differ significantly among the subgroups G1:30.79 (28.35–34.62); G2:29.01 (27.90–30.34); G3:28.79 (26.94–34.91) μmol/L; Kruskal–Wallis *p* = 0.433.

TBARS levels also did not differ significantly among the subgroups G1:5.75 (4.52–6.39); G2:5.02 (3.38–5.84); G3:5.65 (4.47–7.51) μmol/L MDA; Kruskal–Wallis *p* = 0.152. Total antioxidant capacity (TEAC) also did not differ among subgroups G1: 2.345 (2.310–2.408); G2: 2.350 (2.290–2.470); G3: 2.330 (2.258–2.560) mmol/L Trolox; Kruskal–Wallis *p* = 0.910, nor did catalase (CAT) activity G1: 0.137 (0.127–0.183); G2: 0.133 (0.112–0.155); G3: 0.136 (0.108–0.175) U/mg Hb; Kruskal–Wallis *p* = 0.520, glutathione peroxidase (GPx) activity G1: 0.100 (0.089–0.105); G2: 0.097 (0.088–0.108); G3: 0.095 (0.081–0.109) U/mg Hb; Kruskal–Wallis *p* = 0.760, or superoxide dismutase (SOD) activity G1: 0.630 (0.290–1.135); G2: 0.600 (0.400–0.820); G3: 0.595 (0.275–0.870) U/mg Hb; Kruskal–Wallis *p* = 0.679. These results indicate that, although redox imbalance is persistently present in patients with post-COVID-19 syndrome compared to healthy controls, its magnitude does not vary significantly according to the time elapsed since symptom onset, at least within the interval evaluated in this study (up to 32 months).

To further address the possibility of whether the observed redox imbalance was influenced by the unequal sex distribution between groups (Table 1), patients with PCS and controls were compared separately within each sex stratum using the Mann–Whitney U test (Table 3). The pattern of redox imbalance was consistent in both sexes: TBARS, TEAC, CAT, GPx, and SOD concentrations/activities differed significantly between PCS and control participants among both women and men (*p* < 0.05 for all comparisons), with predominantly large effect sizes. Plasma NO levels did not differ significantly between PCS and controls among women (*p* = 0.430) but were significantly lower in the PCS group among men (*p* = 0.039), with a smaller effect size (r = −0.300). Within each study group, biomarker levels were generally similar between women and men, with the exception of catalase activity in the PCS group, which was marginally higher in men (*p* = 0.041), and SOD activity in the control group, which was marginally higher in women (*p* = 0.044); no other marker differed significantly by sex within either group. Taken together, these results indicate that the sex imbalance between groups is unlikely to fully account for the redox differences observed between patients with PCS and healthy controls.

## 4. Discussion

This study investigated the levels of pro-oxidant markers (NO and MDA), enzymatic antioxidant defenses (SOD, CAT, and GPx), and total antioxidant capacity (ABTS) in patients with post-COVID-19 syndrome (PCS) who experienced a mild form of the disease during the acute phase. The results showed that individuals with PCS had elevated pro-oxidant markers and reduced antioxidant defenses compared with healthy controls. Additionally, associations between lipid profile parameters and the oxidative stress markers, MDA and NO, were investigated. Among the PCS patient subgroups, none of the oxidative stress biomarkers, including TBARSs and NO, differed significantly according to symptom duration (Table 2).

A methodological caveat central to the interpretation of these findings concerns the temporal gap between the collection of control samples (2018) and PCS samples (2020–2023). Rather than reflecting years of frozen storage prior to analysis, the redox assays for the control cohort were performed in 2018, contemporaneously with sample collection, using the same standardized protocol later applied to the PCS group; consequently, the differences reported here are not attributable to differential degradation of labile analytes during prolonged frozen storage of either group. Nonetheless, because the two cohorts were not assayed in the same analytical runs, batch-to-batch variability in reagents, calibration, and equipment over this interval cannot be entirely excluded and represents a systematic source of uncertainty that should be considered when interpreting the magnitude of the effect sizes reported below. This limitation is inherent to the use of historical biobank cohorts as controls, an approach also adopted in other studies relating oxidative stress to post-COVID-19 syndrome using pre-pandemic samples [3], and should be borne in mind throughout the interpretation of the present results.

Systemic oxidative stress is considered one of the main mechanisms involved in the pathophysiology of post-COVID-19 syndrome (PCS). This syndrome is associated with a persistent low-grade inflammatory state that favors the activation of nitro-oxidative pathways, resulting in increased production of reactive species and progressive depletion of endogenous antioxidant defenses [3,6,27]. The increase in malondialdehyde (MDA) concentrations suggests sustained lipid peroxidation months after SARS-CoV-2 infection, which is consistent with the findings of the present study. Indeed, patients with PCS in this Amazonian population showed TBARS concentrations approximately three times higher than those of healthy controls 5.490 (4.330–6.950) vs. 1.846 (1.372–2.193) μmol/L MDA; *p* < 0.0001, with a large effect size (r = 0.917), demonstrating that lipid peroxidation remained elevated even in individuals who experienced a mild acute phase and a considerable time interval since infection, given that 61.2% of the sample had symptoms for more than 12 months.

As a product of polyunsaturated fatty acid oxidation, MDA reflects oxidative damage to cell membranes and may compromise the structural and functional integrity of tissues by altering membrane permeability and fluidity, increasing membrane rigidity and dysfunction [12]. This process leads to the loss of membrane receptor function and reduced activity of associated enzymes [28,29]. Furthermore, the breakdown of oxidized lipids generates toxic metabolites, such as MDA and 4-hydroxynonenal (4-HNE), which can cause additional damage to proteins and DNA [12,28]. The persistence of this process can hinder cellular recovery and favor the maintenance of metabolic and inflammatory alterations after the acute phase of the disease [11,30]. In the present study, the increase in MDA observed in the PCS group is consistent with the maintenance of this membrane oxidative damage, supporting the hypothesis that persistent lipid peroxidation contributes to the chronic symptoms reported by patients, particularly fatigue (83.3%), headache (54.8%), and muscle weakness (33.3%), manifestations consistent with impaired tissue integrity.

Elevated levels of lipid peroxidation markers, such as malondialdehyde (MDA/TBARS), are strongly associated with the severity of neuropsychiatric symptoms in patients with long COVID because they represent the activation of neuroaffective toxicity pathways. This process results from a persistent imbalance between the excessive production of reactive species and the exhaustion of antioxidant defenses, such as the enzyme glutathione peroxidase (GPx) [14,31,32]. In this regard, GPx was the marker showing the greatest separation between groups in the present study, exhibiting a marked reduction in activity in the PCS group, with no overlap between groups (r = −1.000; *p* < 0.0001), reinforcing the depletion of this specific antioxidant pathway in these patients.

Lipid peroxidation generates toxic aldehydes (such as MDA) which mediate structural damage. In long COVID, these oxidative stress byproducts cause neuroinflammation and damage to dopaminergic neurons, which is directly linked to the emergence of depression, anxiety, and cognitive deficits [3,33]. The brain is particularly vulnerable to this attack because of its high oxygen consumption and the abundance of fatty acids in its membranes. This vulnerability finds a parallel in the frequency of neurological manifestations observed in the present sample, in which headache (54.8%), insomnia (46.4%), and amnesia (34.5%) were among the most reported symptoms, consistent with the impact of persistent oxidative damage on the central nervous system.

GPx is responsible for degrading lipid hydroperoxides before they cause damage. Studies have shown that patients with severe fatigue and depression symptoms present with chronic GPx deficiency, allowing lipid peroxidation to proceed unchecked, perpetuating the state of cellular toxicity [3,14,31]. This mechanism is consistent with the profile observed in the present study, in which the marked reduction in GPx activity coexisted with elevated TBARS levels and a high prevalence of fatigue (83.3%), reinforcing the link between impairment of this antioxidant defense and symptom persistence in post-COVID-19 syndrome.

In the present study, a marked decrease in superoxide dismutase (SOD), catalase (CAT), and glutathione peroxidase (GPx) activities was observed, all of which were significantly reduced in the PCS group compared with the controls (*p* < 0.0001), with large effect sizes (SOD: r = −0.786; CAT: r = −0.587; GPx: r = −1.000). Failure or deficiency of these primary protective mechanisms is related to oxidative stress, which can result in the oxidation of fundamental biomolecules such as DNA, proteins, and lipids. The decrease in total antioxidant capacity, assessed by the ABTS assay, was also observed in the present sample 2.340 (2.280–2.550) vs. 2.670 (2.620–2.760) mmol/L Trolox; *p* < 0.0001; r = −0.897, corroborating this interpretation and indicating that restoration of redox homeostasis may not be fully achieved in patients affected by post-COVID-19 syndrome [7,13]. Together, the simultaneous depletion of enzymatic and non-enzymatic defenses, accompanied by increased lipid peroxidation, constitutes the pattern of persistent redox imbalance identified in these individuals.

The continuity of these redox alterations is likely associated with the mitochondrial dysfunction observed in patients with long COVID. Recent research indicates that structural and functional mitochondrial alterations may persist after SARS-CoV-2 infection, affecting oxidative phosphorylation and increasing reactive oxygen species generation [34]. These alterations are associated with continuous fatigue, exercise intolerance, and decreased energy production, which are observed in individuals with long COVID [8,13,35].

Acute SARS-CoV-2 infection triggers a continuous state of inflammation and oxidative stress, characterized by the excessive production of reactive oxygen species (ROS) [35]. In long COVID, the persistent immune response increases the demand for antioxidants to neutralize these radicals, resulting in the progressive depletion of endogenous enzymatic reserves. When continuous ROS generation exceeds the body’s antioxidant capacity, a sustained redox imbalance is established, with lasting metabolic and inflammatory consequences [12,35].

The results of the present study are consistent with recent investigations describing persistent oxidative stress in post-COVID-19 syndrome. Studies have shown that individuals with PCS present significantly lower SOD and CAT levels compared with healthy controls [14]; the authors observed increased oxidative biomarkers associated with reduced antioxidant defenses in patients with post-viral fatigue following SARS-CoV-2 infection. Other studies have also demonstrated associations between mitochondrial alterations, bioenergetic disturbances, and maintenance of chronic low-grade inflammation in this condition [8,34,36,37].

The persistence of redox imbalance may affect several physiological systems. In the central nervous system, the prolonged presence of excess reactive oxygen species promotes neuroinflammation, alterations in synaptic homeostasis, and impaired neuronal function, mechanisms frequently related to fatigue and cognitive manifestations observed in chronic fatigue syndrome [6,38]. In the vascular system, oxidative stress facilitates lipoprotein oxidation, decreases nitric oxide bioavailability, and activates endothelial inflammatory pathways, favoring the persistence of vascular dysfunction [39,40].

A plausible unifying mechanism linking these observations is tissue hypoxia secondary to persistent microvascular blood supply reduction. Hypoxia disturbs the balance between reactive oxygen species (ROS) production and antioxidant defenses: prolonged hypoxia stabilizes hypoxia-inducible factors (HIFs) and depletes endogenous antioxidant systems, such as glutathione, leaving tissues vulnerable to oxidative damage, while hypoxia-driven mitochondrial metabolic changes increase ROS generation and drive lipid peroxidation by attacking polyunsaturated fatty acids in cell membranes. This is consistent with the large-magnitude reductions in antioxidant defenses (TEAC/ABTS, SOD, CAT, GPx) and the increase in lipid peroxidation (TBARS) reported here. A previously proposed pathophysiological mechanism for long COVID, involving persistent reduction in microvascular tissue blood supply (blood supply reduction, SR), is compatible with all of the results reported above and reinforces the conceptual model underlying the present study [41]. The SR mechanism leads to tissue hypoxia, which, as discussed above, may explain the redox imbalance observed in this study. Quantitative case–control evidence of microvascular alterations in long COVID has been reported across multiple tissues, including the skin [42], retina [43], bulbar conjunctiva [44], and cerebral gray matter [45], lending further support to a hypoxia-mediated mechanism linking microvascular dysfunction to the persistent redox imbalance described here.

This scenario is supported by evidence that chronic low-grade inflammation and oxidative damage occur simultaneously and feed back on each other in cognitive decline syndromes. In this context, studies conducted in the Brazilian Amazon have demonstrated the persistence of pro-inflammatory cytokines for prolonged periods after the acute phase of SARS-CoV-2 infection, supporting the presence of a chronic inflammatory state that may prolong systemic oxidative damage and favor the continuity of clinical manifestations observed in these individuals [46].

A weak but statistically significant positive correlation was observed between MDA and HDL-c (rs = 0.28; *p* = 0.010), which should be interpreted with caution given its limited strength. This finding does not, by itself, establish a direct biological relationship between lipid peroxidation and HDL-c concentrations, particularly since HDL-c levels did not differ significantly between the PCS and control groups (Table 1); it may instead reflect a broader pattern of persistent oxidative stress in the PCS group, alongside the elevated total cholesterol and LDL-c concentrations observed relative to controls [47]. The lasting mitochondrial alterations after SARS-CoV-2 infection may increase the production of reactive species and impair adenosine triphosphate (ATP) synthesis, potentially contributing to the fatigue and exercise intolerance frequently reported in this cohort [10].

Nitric oxide (NO) levels showed no significant association with any lipid profile parameter (total cholesterol, LDL-c, HDL-c, or triglycerides), indicating that the reduced NO bioavailability observed in this sample occurred independently of lipid concentration. Reduced NO-mediated signaling is associated with persistent endothelial dysfunction and microvascular alterations observed in patients with long COVID [17,48]. Furthermore, elevated triglyceride concentrations may intensify oxidative stress and worsen endothelial function, contributing to the maintenance of vascular damage after infection [49,50].

It should be noted that HDL functionality was not directly assessed in the present study, and the weak MDA-HDL-c correlation observed does not support strong conclusions regarding the antioxidant capacity of HDL in PCS. Under conditions of chronic inflammation, qualitative changes in HDL composition, such as oxidative modification of Apolipoprotein A-I (ApoA-I), have been described elsewhere and could theoretically compromise its protective properties [51,52]; however, this possibility remains speculative in the absence of direct functional measurements in the present sample.

Some limitations must be considered, particularly the small sample size, which may limit the generalizability of the results of this study. In addition, the lack of direct assessment of mitochondrial function precludes the experimental confirmation of the proposed mechanisms. Moreover, the cross-sectional design limits causal inferences between oxidative stress and persistent symptoms. Additionally, the control group consisted of samples from a previous study (2018), which were not contemporaneous with those of the patients, which may introduce pre-analytical variability and differences in storage time for labile analytes such as NOx and erythrocyte enzymatic activity. To minimize this source of variability, samples from both groups were collected, processed, and centrifuged under the same standardized protocol and stored continuously at −80 °C from the time of initial freezing until analysis, without intervening freeze–thaw cycles, which is expected to limit degradation of labile analytes. Nonetheless, the consistency of these results with recent literature data [27] reinforces our proposed interpretation. Furthermore, the PCS and control groups differed significantly in sex distribution (77.65% vs. 45.28% female; *p* = 0.0002). To assess whether this imbalance could account for the observed redox differences, a sex-stratified comparison was performed (see the Results Section and Table 3); the pattern of redox imbalance was consistent in both sexes for most markers, and marker levels did not generally differ by sex within each group, suggesting that the sex imbalance is unlikely to fully explain the group differences. Nonetheless, the smaller male subgroup (*n* = 19) limits statistical power for this stratified analysis relative to the female subgroup, and residual confounding by sex and hormonal status cannot be entirely excluded. In addition, patients with PCS were found to be significantly younger than controls (Table 1), and age-related changes in antioxidant defenses and lipid metabolism cannot be entirely ruled out as a contributing factor to the group differences observed, alongside the sex imbalance discussed above. It is also important to highlight that the sample included individuals who experienced a mild acute phase, indicating that the redox imbalance observed may occur even in the absence of severe initial disease.

The results corroborate the importance of oxidative stress in the pathophysiology of post-COVID-19 syndrome. The increase in lipid peroxidation, together with the decrease in antioxidant defenses, indicates the maintenance of a continuous oxidative state, possibly linked to the inflammatory, metabolic, and vascular alterations identified after SARS-CoV-2 infection. The reduced nitric oxide bioavailability observed in the PCS group, although showing no significant variation with disease duration, is consistent with the vascular component of this imbalance. Future research focused on functional mitochondrial assessment, as well as on more specific redox markers, may further the understanding of these mechanisms and aid in the development of targeted therapeutic strategies.

## 5. Conclusions

Patients with post-COVID-19 syndrome show sustained oxidative alterations even after the acute phase of infection, characterized by increased lipid peroxidation and reduced antioxidant defenses. The reduction in NO levels in the PCS group relative to the controls is consistent with the possible involvement of vascular alterations in long COVID, although no significant variation was observed according to disease duration, which requires confirmation in longitudinal studies. These findings reinforce the presence of continuous metabolic impairment after SARS-CoV-2 infection in the Amazonian population.

## Figures and Tables

**Table 1:** The sociodemographic, laboratory, and clinical characteristics of the participants included in the study.

Variables	Long COVID Group (*n* = 85)	Control Group (*n* = 53)	*p*- Value
Sex			
Female, *n* (%)	66 (77.65%)	24 (45.28%)	0.0002 ***
Age, *n*, median (Q1–Q3), years	*n* = 85, 49 (42–60)	*n* = 53, 55 (47–62)	0.035
Laboratory results			
Fasting glucose (*n*) median (Q1–Q3)	(*n* = 85) 94 (88–100)	95 (87–105)	0.887
Total cholesterol, (*n*) median (Q1–Q3)	(*n* = 85) 201 (179–235)	174 (157–194)	0.0001
HDL-c, (*n*) median (Q1–Q3)	(*n* = 85) 50 (44–59)	52 (46–57)	0.423
LDL-c, (*n*) median (Q1–Q3)	(*n* = 85) 123 (101–153)	105.5 (86–119.25)	0.0012
Triglycerides, (*n*) median (Q1–Q3)	(*n* = 85) 124 (84–175)	134 (94–170)	0.506
Long COVID symptoms (*n* = 84/85)			
Fatigue, *n* (%)	70 (83.33%)		
Myalgia, *n* (%)	34 (40.48%)		
Insomnia, *n* (%)	39 (46.43%)		
Muscle weakness, *n* (%)	28 (33.33%)		
Dyspnea, *n* (%)	31 (36.90%)		
Chest pain, *n* (%)	30 (35.71%)		
Headache, *n* (%)	46 (54.76%)		
Cough, *n* (%)	16 (19.05%)		
Amnesia, *n* (%)	29 (34.52%)		
Duration of Long COVID	(*n* = 85)		
≤6 months, *n* (%)	12 (14.12%)		
>6–12 months, *n* (%)	21 (24.71%)		
>12 months, *n* (%)	52 (61.18%)		

Continuous data are presented as the mean ± standard deviation (SD), and categorical data are presented as absolute frequency and percentage (*n* [%]). Between-group comparisons were performed using the Mann–Whitney U test for continuous variables and the chi-square test for categorical variables. *** *p* < 0.001. Statistical significance was set at *p* < 0.05. HDL-c: high-density lipoprotein cholesterol; LDL-c: low-density lipoprotein cholesterol; SD: standard deviation. Partial *n* indicates the number of patients with available data for the variable.

**Table 2:** Oxidative stress biomarkers according to long COVID duration.

Biomarker	Unit	G1 ≤ 6 Months	G2 > 6–12 Months	G3 > 12 Months	G1 vs. G2	G1 vs. G3	G2 vs. G3
NO	µmol/L	30.79 (28.35–34.62)	29.01 (27.90–30.34)	28.79 (26.94–34.91)	ns	ns	ns
TBARS	µmol/L	5.75 (4.52–6.39)	5.02 (3.38–5.84)	5.65 (4.47–7.51)	ns	ns	ns
TEAC/Trolox	mmol/L	2.345 (2.310–2.408)	2.350 (2.290–2.470)	2.330 (2.258–2.560)	ns	ns	ns
CAT	U/mg Hb	0.137 (0.127–0.183)	0.133 (0.112–0.155)	0.136 (0.108–0.175)	ns	ns	ns
GPx	U/mg Hb	0.100 (0.089–0.105)	0.097 (0.088–0.108)	0.095 (0.081–0.109)	ns	ns	ns
SOD	U/mg Hb	0.630 (0.290–1.135)	0.600 (0.400–0.820)	0.595 (0.275–0.870)	ns	ns	ns

Data are presented as medians (Q1–Q3). Subgroup comparisons were performed using the Kruskal–Wallis test. There were no significant differences among the subgroups for any marker (Kruskal–Wallis: TBARS *p* = 0.152; NO *p* = 0.433; TEAC *p* = 0.910; SOD *p* = 0.679; GPx *p* = 0.760; CAT *p* = 0.520); therefore, pairwise comparisons (Dunn’s post hoc test) were not performed and are presented as ns (not significant). NO: nitric oxide; TBARS: thiobarbituric acid reactive substance; TEAC: Trolox equivalent antioxidant capacity; CAT: catalase; GPx: glutathione peroxidase; SOD: superoxide dismutase.

**Table 3:** Oxidative stress biomarkers according to sex, stratified within the post-COVID-19 syndrome (PCS) and control (HC) groups.

Biomarker	Unit	Female PCS	Female HC	*p* (F)	Male PCS	Male HC	*p* (M)
NO	µmol/L	28.920 (27.240–34.522)	29.673 (27.455–35.494)	0.430	29.460 (26.015–33.785)	32.999 (29.229–37.435)	0.039
TBARS	µmol/L	5.526 (4.548–7.269)	1.858 (1.688–2.414)	<0.0001	5.274 (3.255–6.078)	1.740 (1.215–2.130)	<0.0001
TEAC/Trolox	mmol/L	2.330 (2.270–2.525)	2.650 (2.610–2.692)	<0.0001	2.350 (2.305–2.575)	2.720 (2.620–2.790)	<0.0001
CAT	U/mg Hb	0.133 (0.109–0.161)	0.350 (0.282–0.412)	<0.0001	0.156 (0.131–0.177)	0.280 (0.190–0.390)	0.013
GPx	U/mg Hb	0.095 (0.085–0.107)	0.460 (0.370–0.575)	<0.0001	0.101 (0.091–0.112)	0.400 (0.340–0.490)	<0.0001
SOD	U/mg Hb	0.600 (0.300–0.865)	2.425 (1.720–2.652)	<0.0001	0.560 (0.285–1.050)	1.420 (1.020–2.210)	<0.0001

Data are presented as medians (Q1–Q3). Comparisons between PCS and healthy control (HC) participants were performed separately within each sex stratum using the Mann–Whitney U test. NO: nitric oxide; TBARS: thiobarbituric acid reactive substance; TEAC: Trolox equivalent antioxidant capacity; CAT: catalase; GPx: glutathione peroxidase; SOD: superoxide dismutase.

## Data Availability

All data are contained in the article.
